# Optimization of the Multi-Channel Surface Electrogram Acquisition for Noninvasive Uterine Electrophysiology Imaging

**DOI:** 10.1007/s10439-026-04072-2

**Published:** 2026-04-10

**Authors:** Hansong Gao, Sicheng Wang, Yiqi Lin, Meng Jiang, Yuelin Li, Qiuchang Sun, Zichao Wen, Yong Wang

**Affiliations:** 1 Department of Electrical and Systems Engineering, Washington University in St. Louis, St. Louis, MO 63130 USA; 2 Department of Obstetrics & Gynecology, Washington University in St. Louis School of Medicine, St. Louis, MO 63110 USA; 3 Department of Biomedical Engineering, Washington University in St. Louis, St. Louis, MO 63130 USA; 4 Mallinckrodt Institute of Radiology, Washington University in St. Louis School of Medicine, St. Louis, MO 63110 USA

**Keywords:** Noninvasive electrophysiology imaging, Electrode placement, Inverse problem, Optimization, Uterine peristalsis imaging

## Abstract

**Purpose:**

The goal of optimizing electrogram acquisition in electrophysiology imaging is to minimize the number of electrodes used, determine their optimal placement, and ensure accurate imaging.

**Methods:**

We quantitatively evaluated the impact of the electrode number and placement on the accuracy of human uterine peristalsis imaging (UPI) using adapted minimum nonzero eigenvalue pursuit (MNEP), adapted maximal projection on minimum eigenspace (MPME), and a distance-based baseline method. Specifically, we assessed the accuracy of the uterine surface electrogram imaged by UPI while maintaining the original 128-electrode UPI placement as a constraint. Subsequently, we expanded our analysis to the entire body surface of the subjects, removing the constraint of the initial UPI electrode placement to explore a generalized optimized electrode placement strategy.

**Results:**

The MPME method demonstrated superior performance compared to other approaches under both constrained and unconstrained optimization. For UPI, the optimal electrode configuration should prioritize a dense concentration of electrodes on the lower abdomen, close to the uterus, where the most informative bioelectrical signals are captured. At the same time, it is important to maintain a distribution of electrodes along the edges of the upper abdominal, lateral sides, and posterior regions. This spatial distribution preserves the geometric contour of the torso and supports comprehensive, multi-angle observation of uterine electrical activity. By combining signal fidelity with spatial coverage, this balanced placement strategy enhances the UPI accuracy.

**Conclusions:**

The adapted MPME method guided a novel electrode placement strategy for electrophysiological imaging, achieving substantial improvements in imaging accuracy while reducing system complexity and cost.

**Supplementary Information:**

The online version contains supplementary material available at 10.1007/s10439-026-04072-2.

## Introduction

In recent years, noninvasive electrophysiology imaging systems have significantly advanced the field of electrophysiology by enabling detailed imaging and visualization of electrophysiological activities in various electrically excitable organs without invasive procedures. Among these systems, electrocardiographic imaging (ECGI) of the human heartbeat, electromyometrial imaging (EMMI) of the uterine contractions during human labor, and uterine peristalsis imaging (UPI) of the nonpregnant human uterine peristalsis have been developed and translated into human applications [1–6]. Specifically, ECGI is a noninvasive functional imaging technique used to image cardiac arrhythmias and define the electrophysiologic properties of the entire heart [7, 8]. This method allows for detailed noninvasive cardiac electrical activity mapping, reducing the need for invasive procedures and associated risks. EMMI was designed to noninvasively monitor detailed 3D uterine contraction patterns across the entire uterine surface during human labor, offering a safe and effective tool for studying uterine activity without posing any risk to the fetus [9, 10]. UPI is designed to noninvasively quantify the 3D electrical activation patterns of uterine peristalsis during the menstrual cycle, offering valuable insights into uterine disorders [11–16]. Although electrophysiological imaging techniques such as UPI involve a higher cost than conventional surface electrohysterography (EHG), this difference is primarily driven by the larger number of electrodes and the need for subject-specific geometry acquisition. In return, UPI provides unique spatiotemporal resolution and full-surface coverage of uterine electrical activity that conventional low-channel EHG cannot achieve. As a comparison, UPI remains far more cost-effective and practical compared with popular medical imaging modalities, such as functional magnetic resonance imaging (fMRI) or positron emission tomography (PET), which typically cost thousands of dollars per scan. By contrast, electrophysiological imaging techniques require only a short MRI session (on the order of a hundred dollars) to acquire subject-specific body-uterus geometry.

All these electrophysiological imaging systems share a similar procedure involving three key steps: acquiring a subject-specific body-organ geometry via MRI or CT scan, measuring multiple-channel body surface electric potentials with unipolar electrodes, and solving the three-dimensional Cauchy problem to reconstruct the organ surface potentials [17, 18]. The performance of these systems is highly dependent on the number and placement of electrodes, which are critical for accurately capturing temporal-spatial bioelectric fields. However, these systems share common practical challenges related to electrode placement. For example, ECGI typically requires many electrodes (150-250) to be placed on the body, potentially complicating the clinical application due to the effort and time necessary for the precise placement. EMMI faces more significant challenges with electrode placement on the abdominal surface of a laboring woman, which can be cumbersome and potentially interfere with intense clinical care during labor. Similarly, placing over 100 electrodes around a nonpregnant patient’s body surface during their gynecological visit can be troublesome and prevent UPI's clinical adoption.

Current strategies of electrode placement lack systematic optimization, leading to suboptimal imaging quality and increased costs due to excessive electrode use. Previous studies used fixed electrode configurations to investigate the effects of electrode number and placement for electrophysiology imaging applications, but these approaches were not systematically optimized [19, 20]. Given these limitations, there is a growing need to improve electrode placement strategies guided by systematic optimization. The objective is to reduce the number of electrodes and optimize placement without compromising imaging quality, thereby decreasing costs and simplifying the experimental setup. More flexible and configurable electrode patches could enhance the accuracy and efficiency of these noninvasive electrophysiology imaging systems. In this work, we focus on optimizing electrode placement in UPI. Insights can be extended to other noninvasive electrophysiology imaging systems, such as ECGI and EMMI.

To determine the optimal electrode placement configuration, the most straightforward method is to evaluate the performance of all possible combinations of electrode placement. However, a combination approach is computationally intractable if the electrode number is large. Existing methods tackling this problem can be generally divided into two categories: convex relaxation and heuristic algorithms. The convex relaxation method addresses the electrode placement by converting a nonconvex optimization into a convex one through the relaxation of the Boolean constraint [21]. Local optimization techniques can further enhance the accuracy of these results. However, the application of convex relaxation is limited due to the computational complexity. On the other hand, there are heuristic algorithms, such as framesense, minimum nonzero eigenvalue pursuit (MNEP), and maximal projection on minimum eigenspace (MPME) [22, 23]. Framesense aims to minimize mean square error (MSE) by minimizing frame potential. Both MNEP and MPME aim to optimize MSE by maximizing the minimum eigenvalue. These heuristic methods are better suited for large-scale problems because of their computational efficiency.

For the problem formulation in UPI and other electrophysiology imaging systems (EMMI for the pregnant uterus, ECGI for the heart), the number of imaging parameters exceeds the number of electrodes, resulting in an underdetermined linear inverse problem [24]. In addition, we solve the Cauchy problem for Laplace’s equation with Dirichlet and Neumann conditions to reconstruct the bioelectric field. The submatrix corresponding to the Neumann boundary condition in the system matrix must be pre-selected. Therefore, the electrode optimization method should be able to take these two requirements into account. First, it has to be adaptable to an underdetermined problem. Second, it has to ensure that adding new sensing locations does not affect the preselected submatrix. Framesense is a greedy method that iteratively removes the rows in the system matrix, causing the greatest increase in the frame potential at each step. It cannot guarantee that the preselected submatrix is unaffected. For MNEP and MPME, both methods require more electrodes than imaging parameters to form an overdetermined linear inverse problem. However, by modifying the loss metric (LM) and initialization, MNEP and MPME can be adapted to solve the electrode placement problem for UPI.

In this study, we utilized MNEP and MPME to evaluate the impact of the number of electrodes and placement on UPI accuracy. The remainder of this paper is structured as follows: "Materials and Methods" section provides an overview of the data collection, forward computation, the underdetermined linear inverse problem in UPI, electrode optimization methods, and the statistical tools used to evaluate imaging quality. We initially evaluated different electrode optimization methods constrained by the original 128-electrode UPI placement, using it as the gold standard. Subsequently, we extend the analysis by removing this constraint, allowing for a more general evaluation of electrode placement across the entire body geometry. In "Results" section, we quantitatively compare three electrode optimization methods in human subjects and evaluate the prioritized location of electrodes both with and without the constraint. "Discussion" section concludes our findings, discusses the limitations of the current approach, and explores how electrode placement may influence future UPI studies and related techniques.

## Materials and Methods

### Data Collection for UPI

The UPI study was approved by the Washington University Institutional Review Board and was performed in compliance with their guidelines and regulations. The study cohort consisted of 18 subjects, including 10 healthy volunteers and 8 with underlying conditions. Menstrual cycle status was tracked, and all data were collected during the menses phase. Demographic characteristics of the subjects are summarized in Table 1.

**Table 1:** Demographic characteristics of enrolled subjects

Demographic parameters	Value (mean ± SD, range)
Age (years)	28.4 ± 4.0 (21–34)
Body mass index (kg/m^2^)	26.5 ± 4.4 (19.8–34.45)
Race, *n* (%)	
White	11 (61.1%)
Black	6 (33.3%)
Asian	1 (5.6%)
Health status, *n* (%)	
Healthy	10 (55.6%)
Ovulatory dysfunction	2 (11.0%)
Nexplanon	3 (16.7%)
Endometriosis	3 (16.7%)

**Fig. 1:**
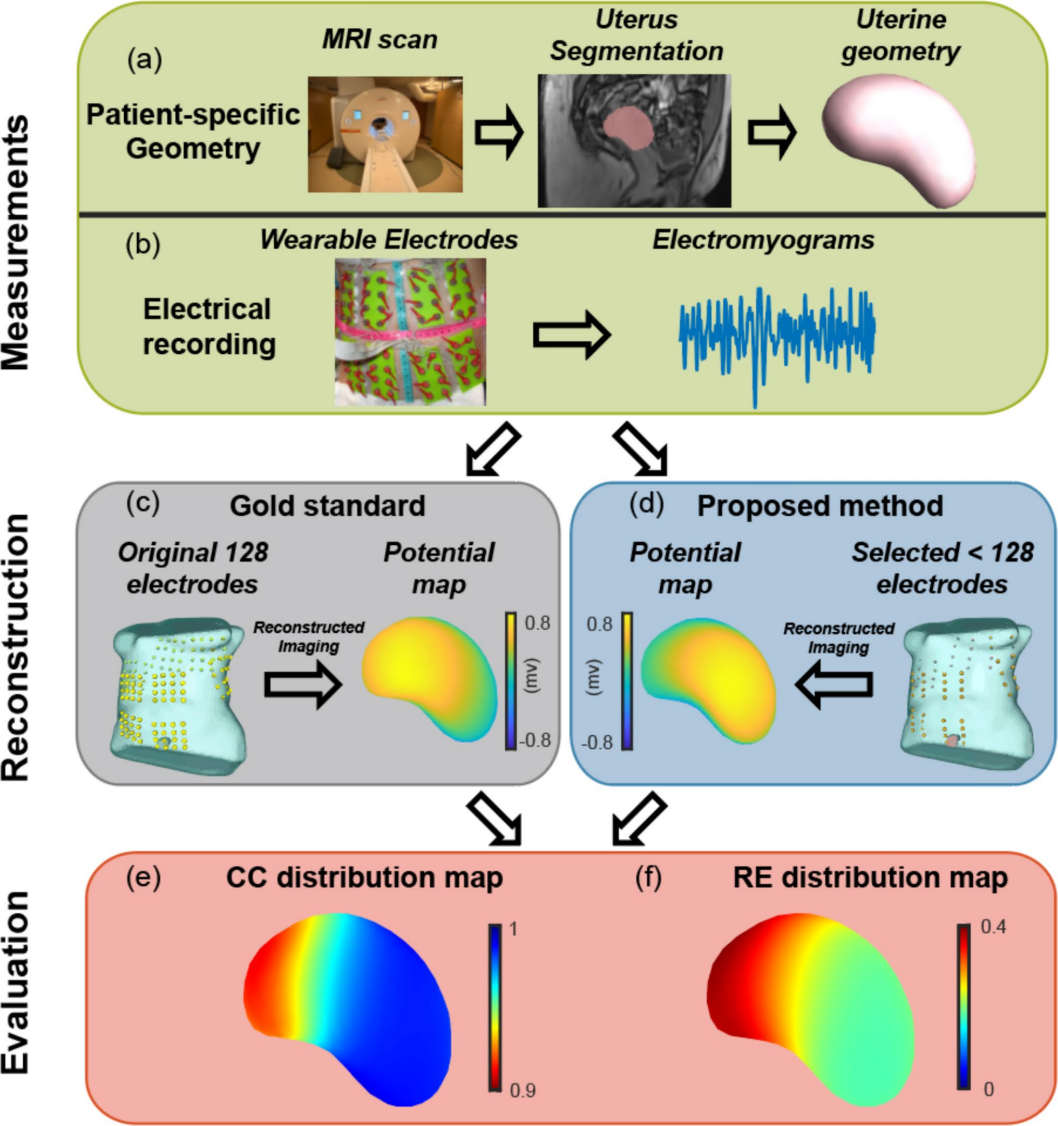
UPI data acquisition, reconstructed imaging, and electrode placement evaluation. **a** Uterine geometry was acquired from MRI images. **b** Body surface electromyograms are recorded from 128 pin-type unipolar electrodes. **c** Gold standard potential map was computed using all 128 electrodes. **d** Potential map reconstructed from selected electrodes (number of electrodes *n* < 128). **e** Correlation coefficients (CC) distribution map across the entire uterine surface between the gold standard potential map and the potential map reconstructed using selected electrodes. **f** Relative errors (RE) distribution map across the entire uterine surface between the gold standard potential map and the potential map reconstructed using selected electrodes. UPI, uterine peristalsis imaging

Each subject underwent MRI (Fig. 1a), and the resulting images were segmented to generate three-dimensional meshes representing uterine geometry. Electrode coordinates were registered to the MRI-derived uterine geometry, enabling construction of a subject-specific body-uterus geometry in a common 3D coordinate system.

Electrophysiological recordings were collected using a high-density array of 128 unipolar, pin-type active electrodes (BioSemi ActiveTwo system, BioSemi B.V., Amsterdam, The Netherlands) positioned on the body surface (Fig. 1b). Electrode placement was guided by anatomical landmarks to ensure consistency across subjects, with detailed specifications provided in the Supplementary section. Signals were sampled at 2048 Hz, bandpass filtered with a Butterworth filter (0.01-0.1 Hz) to preserve uterine activity as suggested by previous studies [6, 25], and downsampled to 1 Hz.

For quality control, channels with poor signal quality were excluded, with a maximum of five channels removed per subject. Specifically, channels with peak amplitudes < 0.01 mV were considered to reflect poor electrode connectivity, whereas those exceeding 2 mV were considered unlikely to be of physiological origin and were attributed to motion or non-physiological artifacts.

The final dataset, comprising body-uterus geometry and around 30 min of electrical recordings for each subject, was used to develop and validate the electrode optimization methods.

### The UPI System

In the UPI system, the inductive effects of the volume conductor are considered negligible, leading to the electro-quasi-static (EQS) condition [17, 26]. The EQS approximation applies at sufficiently low frequencies, typically well below 1 MHz, where inductive and propagation effects can be ignored [27]. Given that our frequency range of interest is 0.01-0.1 Hz, the quasi-static condition is also valid in our system. Under this assumption, the objective of the bioelectric field computation is to reconstruct the electric potential across the entire 3D uterine surface by combining the subject-specific geometry of the uterus and body surface with the electric potential measured on the body surface. The forward problem is defined by relating the uterine surface potential to the potential measured on the body surface through a system matrix by solving the Cauchy problem for Laplace’s equation with Dirichlet and Neumann conditions on the body surface using the method of fundamental solution (MFS) [17]. The relation between body surface potential and uterine surface potential is described by

1 $$\begin{array}{c}{\phi }_{B}={A \phi }_{U}\end{array}$$

where ${\phi }_{B}$ represents the body surface potential, ${\phi }_{U}$ represents the uterine surface potential, and $A$ represents the system matrix.

To optimize the system, we denote

2 $$\begin{array}{c}L\left(s\right)=\mathit{tr}\left({\left(\Psi +\alpha I\right)}^{-1}\right)={\sum }_{k=1}^{n}\frac{1}{{\lambda }_{rk}} \end{array}$$

as the loss metric (LM), where $s$ represents the selected set of electrodes and $\alpha$ is a small constant ($\alpha$ = 0.1 in this study),$\Psi = {A}^{T}A$, ${\lambda }_{r1}\ge {\lambda }_{r2}\ge \dots {\lambda }_{rn}$ are the eigenvalues of $\Psi +\alpha I$.

Here, we provide a concise description of the UPI system; more detailed mathematical derivations are available in the Supplementary section.

### UPI Electrode Optimization Methods

We compared three electrode optimization methods: MPME, MNEP, and the distance-based baseline method.

To describe the electrode optimization process, we introduce $\widetilde{A }={\left[{\widetilde{A}}_{a}, {\widetilde{A}}_{b}\right]}^{T}\in {\mathbb{R}}^{2N\times M}$ corresponding to the original *N* electrode matrix. ${\widetilde{A}}_{a}$ is the submatrix of $\widetilde{A}$ corresponding to the Dirichlet boundary condition, where each row corresponds to a sensing location of an electrode on the subject’s body surface. ${A}_{a}\in {\mathbb{R}}^{n\times M} (n<N)$ is the matrix corresponding to the $n$ electrodes selected from the original *N* electrodes. ${\widetilde{A}}_{b}$ corresponds to the Neumann boundary condition, which is not related to the body surface electric potential measurements but a preselected submatrix of the $A$. ($A={\left[{A}_{a}, {\widetilde{A}}_{b}\right]}^{T}$). Therefore, the initialization of the electrode selection for the UPI problem starts with a preselected submatrix ${\widetilde{A}}_{b}$. Let ${{\varphi }_{k}}^{T}$ be $k$ th selected sensing location in ${\widetilde{A}}_{a}$.${A}_{ak}$ is a submatrix of ${\widetilde{A}}_{a}$, including the first $k$ sensing locations ${A}_{ak}={\left[{{{\varphi }_{k},A}_{a(k-1)}}^{T}\right]}^{T} \in {\mathbb{R}}^{k\times N}$. We denote ${A}_{k}=[{A}_{ak}, {\widetilde{A}}_{b}{]}^{T}$ and ${\Psi }_{k}={A}_{k}^{T}{A}_{k}$. Then, the electrode optimization is transformed into selecting a proper row ${{\varphi }_{k}}^{T}$ to optimize the $L\left(s\right)$ at each step [23].

Both MPME and MNEP are greedy methods that select a new sensing location at each step. The MNEP algorithm selects the sensing location ${{\varphi }_{{s}_{k}}}^{T}$ that maximizes the minimum nonzero eigenvalue of ${\Psi }_{k}$ at each step:

3 $$\begin{array}{c}{s}_{k}= {\underset{i}{argmax} \lambda_{k}} ({A}_{k-1}^{T}{A}_{k-1}+{\varphi }_{i}{\varphi }_{i}^{T})\end{array}$$

where ${s}_{k}$ denotes the selected sensing location at the *kth* step, ${\lambda }_{k}$ denotes the *kth* eigenvalue, which is the nonzero minimum eigenvalue of ${\Psi }_{k}$ at the *kth* step, ${\varphi }_{i}$ denotes the set of all the available candidate rows at the *kth* step.

For the MPME algorithm, the *kth* selected sensing location ${{\varphi }_{{s}_{k}}}^{T}$ has the largest projection on the minimum eigenspace of ${\Psi }_{k-1}$:

4 $$\begin{array}{c}{s}_{k}= \underset{i}{\mathrm{argmax}}||{P}_{k-1}{\varphi }_{i}{||}_{2}^{2}\end{array}$$

where ${s}_{k}$ denotes the selected sensing location at the *kth* step, ${P}_{k-1}$ denotes the projection matrix corresponding to the minimum eigenspace of ${\Psi }_{k-1}$ , and ${\varphi }_{i}$ denotes the set of all the available candidate rows at the *kth* step.

For the distance-based baseline method, the importance of each electrode is ranked based on its Euclidean distance to the centroid of the uterus. The centroid of the uterus is computed by

5 $$\begin{array}{c}{x}_{c}= \frac{{x}_{1}+{x}_{2}+\dots +{x}_{n}}{n},{y}_{c}= \frac{{y}_{1}+{y}_{2}+\dots +{y}_{n}}{n},{z}_{c}= \frac{{z}_{1}+{z}_{2}+\dots +{z}_{n}}{n} \end{array}$$

where ${x}_{c},{y}_{c},{z}_{c}$ denote the coordinates of the centroid of the uterus, ${x}_{n},{y}_{n},{z}_{n}$ denote the coordinates of the mesh points of the uterus.

The Euclidean distance is computed by

6 $$\begin{array}{c}d=\sqrt[2]{{\left({x}_{e}-{x}_{c}\right)}^{2}+{\left({y}_{e}-{y}_{c}\right)}^{2}+{\left({z}_{e}-{z}_{c}\right)}^{2}}\end{array}$$

where ${x}_{c},{y}_{c},{z}_{c}$ denote the coordinates of the centroid of the uterus, ${x}_{e},{y}_{e},{z}_{e}$ denote the coordinates of the new sensing location.

The method selects a new sensing location that is closest to the centroid of the uterus at each step.

### Evaluate Electrode Optimization Methods under the Constraint of Original UPI Electrode Placement

Uterine surface electrograms reconstructed from the original 128-electrode placement on the subject’s body surface serve as the gold standard for this evaluation (Fig. 1c). We compared the effectiveness of three electrode optimization methods, MPME, MNEP, and the distance-based baseline method, in reconstructing electrograms at 320 uterine sites. Reconstruction imaging accuracy was evaluated by reducing the number of electrodes from 112 to 64 in steps of 16 (Fig. 1d). This analysis validated electrode optimization methods and identified specific electrode sensing locations that significantly affect reconstructed imaging accuracy. The error of reconstructed electrograms caused by reducing the number of electrodes is evaluated by computing relative error (RE, which quantifies amplitude differences) and correlation coefficient (CC, which quantifies morphological differences), defined as follows [4]:

7 $$\begin{array}{c}RE=\sqrt{\frac{{\sum }_{i=1}^{n}{\left({X}_{i}^{RD}-{X}_{i}^{OG}\right)}^{2}}{{\sum }_{i=1}^{n}{\left({X}_{i}^{OG}\right)}^{2}}}\end{array}$$

8 $$\begin{array}{c}CC=\frac{{\sum }_{i=1}^{n}{(X}_{i}^{OG}-\overline{{X }^{OG}})({X}_{i}^{RD}-\overline{{X }^{RD}})}{\sqrt{{\sum }_{i=1}^{n}{\left({X}_{i}^{OG}-\overline{{X }^{OG}}\right)}^{2}}\sqrt{{\sum }_{i=1}^{n}{\left({X}_{i}^{RD}-\overline{{X }^{RD}}\right)}^{2}}}\end{array}$$

Temporal CC (Fig. 1e) and RE (Fig. 1f) were calculated at each uterine site. Here, $X$ denotes potential, ${X}_{i}^{OG}$ represents the potential for the *ith* frame reconstructed from the original 128 electrodes and ${X}_{i}^{RD}$ represents the potential for the *ith* frame reconstructed from a reduced number (*n* < 128) of electrodes. $\overline{{X }^{OG}}$ and $\overline{{X }^{RD}}$ are the average potential of all the time frames reconstructed from the original and reduced number of electrodes.

To assess the performance of the three electrode optimization methods, we conducted comparisons at the uterine site, subject, and group levels. For the uterine site level comparison, we computed the CC and RE at one representative uterine site with different numbers of electrodes. For the subject level comparison, we investigated the accuracy and robustness of electrode optimization methods with varying numbers of electrodes using subject #1 as an example. We first generate spatial distribution maps of CC and RE to visualize the reconstructed imaging accuracy across the entire uterine surface. After that, quantitative analyses of CC and RE were performed with the number of electrodes reduced from 112 to 64 in steps of 16. To further validate the effectiveness of the three electrode optimization methods and ensure their generalizability, we expanded our analysis to include UPI data of 18 subjects at the group level. Each subject’s UPI utilizes MRI-derived subject-specific geometry, ensuring that electrode placement, as well as body and uterine geometries, are tailored to the subject. We evaluated the three different electrode optimization methods by performing CC and RE analyses using a 64-electrode UPI compared to the 128-electrode UPI in all 18 subjects. To determine the significance of the difference observed between different electrode selection methods at the group level, a nonparametric Wilcoxon signed-rank test was employed to compare the mean values of CC and RE of each subject for three electrode selection methods, since the differences between paired data are not normally distributed. The resulting *p* values were compared against a significance level of 0.05 to determine statistical significance. We also reported the Hodges–Lehmann (HL) estimator of the median paired difference along with its 95% confidence interval (obtained by bootstrap resampling). To quantify the magnitude of the effect, we calculated the effect size as $r=Z/\sqrt{N}$, where $N$ is the number of non-zero pairs. The statistical analysis was performed using MATLAB (2022b). In addition, we computed the LM for each case to demonstrate that optimizing the LM results in higher CC and lower RE.

### Evaluate the Prioritized Electrode Locations under the Constraint of the Original UPI Placement

In the results section, the MPME method demonstrated superior performance compared to the other two methods. Therefore, we evaluated the prioritized electrode location under the constraint of the original 128-electrode placement using the MPME method. Not all 128 electrodes contribute equally to the accuracy of reconstructed imaging. Identifying the most critical electrode locations is essential for optimizing UPI electrode placement. In MPME, the electrodes are sequentially selected, so those chosen earlier are considered more important. We rank the importance of each electrode based on this selection order. To illustrate this prioritization, we present electrode placement results from three representative subjects.

### Evaluate the Prioritized Electrode Locations without the Constraint of the Original UPI Placement

After evaluating the electrode optimization methods under the constraint of the original 128-electrode UPI placement, we extended our analysis to the entire body surface to guide a new design of electrode placement. Since all subjects share a similar body-uterus geometry, we used subject #1 to demonstrate the analysis. We first defined an accessible body-surface region for electrode placement based on practical and anatomical considerations (Fig. 7a). The accessible region includes the anterior abdominal surface below the breasts and above the pubic region, the lateral abdominal flanks, and the posterior surface above the hip bone. Regions that are impractical for electrode placement or electrically unfavorable, including private areas and bony regions, such as the hips and spine, were excluded, as these areas are either inaccessible during labor or have poor electrical conductivity. Importantly, this definition relies on external anatomical landmarks and does not require prior knowledge of uterine location from MRI.

Each UPI electrode covers a circular region with a diameter of 2 cm. Based on this, the accessible regions on the body surface were discretized into 400 sensing locations (mesh in Fig. 7a). The MPME method was then implemented to select 128 sensing locations. Optimizing the LM results in an improved correlation coefficient (CC) and a reduced relative error (RE). Due to the unavailability of signal data for computing the correlation coefficient (CC) and relative error (RE), we evaluated the performance by comparing the LM of the system matrices for both the original UPI placement and MPME-designed placement.

To enable a quantitative and reproducible description of electrode distribution, we further subdivided the accessible surface into five regions: anterior upper, anterior lower, lateral, posterior upper, and posterior lower (Fig. 7a). Let $y$ denote the anterior-posterior coordinate (with increasing $y$ pointing posteriorly) and $z$ denote the superior-inferior coordinate. Anterior and posterior bands were defined using normalized thresholds along the $y$-axis: vertices with

9 $$\begin{array}{c}y<{y}_{min}+0.25\left({y}_{max}-{y}_{min}\right)\end{array}$$

were classified as anterior, vertices with

10 $$\begin{array}{c}y>{y}_{max}-0.25\left({y}_{max}-{y}_{min}\right)\end{array}$$

were classified as posterior, and the remaining vertices as lateral. Within the anterior and posterior regions, upper and lower subregions were defined separately using the midpoint of the $z$-range of each band. Specifically, anterior vertices with $z$ greater than the anterior band midpoint were labeled anterior upper, and those below were labeled anterior lower. An analogous rule was applied to define posterior upper and posterior lower regions.

Each electrode was assigned to a region based on the region label of its nearest surface vertex, and electrode allocation was summarized as the percentage of electrodes in each region.

## Results

### Evaluation of Electrode Optimization Methods under the Constraint of the Original UPI Placement

**Fig. 2:**
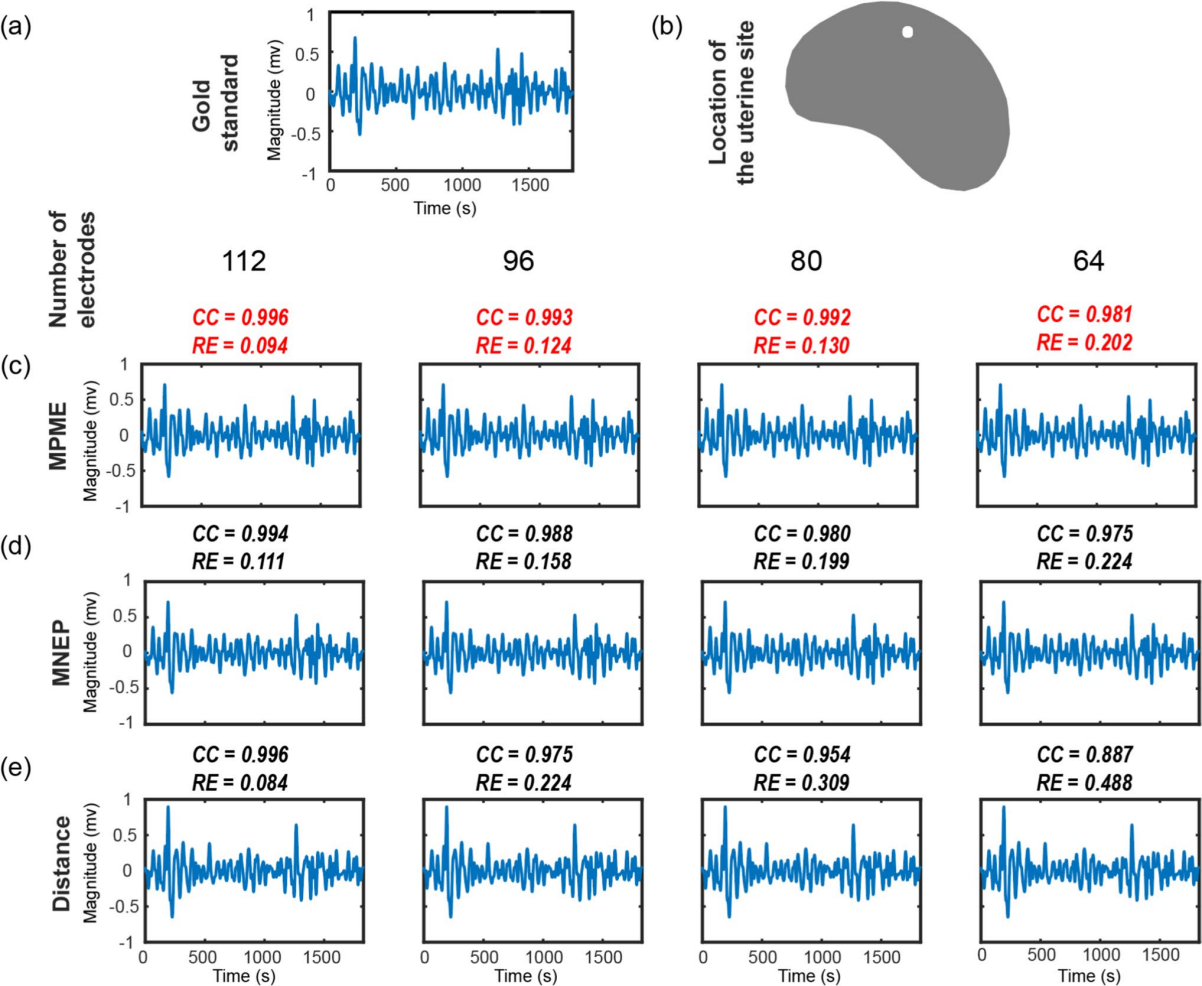
Comparison of reconstructed uterine surface electrograms from a representative uterine site using different electrode optimization methods with varying numbers of electrodes. **a** Gold standard uterine surface electrograms with 128 electrodes. **b** Location of the representative uterine site. **c** Reconstructed uterine surface electrograms using the MPME method with 112, 96, 80, and 64 electrodes. **d** Reconstructed uterine surface electrograms using the MNEP method with 112, 96, 80, and 64 electrodes. **e** Reconstructed uterine surface electrograms using the distance-based method with 112, 96, 80, and 64 electrodes. Red fonts indicate the highest CC and lowest RE. CC, correlation coefficient; RE, relative error; MPME, maximal projection on minimum eigenspace. MNEP, minimum nonzero eigenvalue pursuit

In this section, we first used a uterine surface electrogram from a representative uterine site of subject #1, as shown in Fig. 2a, as an example. We then extended the analysis to the subject and group levels. Uterine surface electrograms were reconstructed using 112 to 64 electrodes for subject #1, as shown in Fig. 2c-e. For the MPME method, the reconstructed uterine surface electrograms demonstrated the highest accuracy with 112 electrodes (CC = 0.996, RE = 0.094), 96 electrodes (CC = 0.993, RE = 0.124), 80 electrodes (CC = 0.992, RE = 0.130), and 64 electrodes (CC = 0.981, RE = 0.202), as shown in Fig. 2c.

**Fig. 3:**
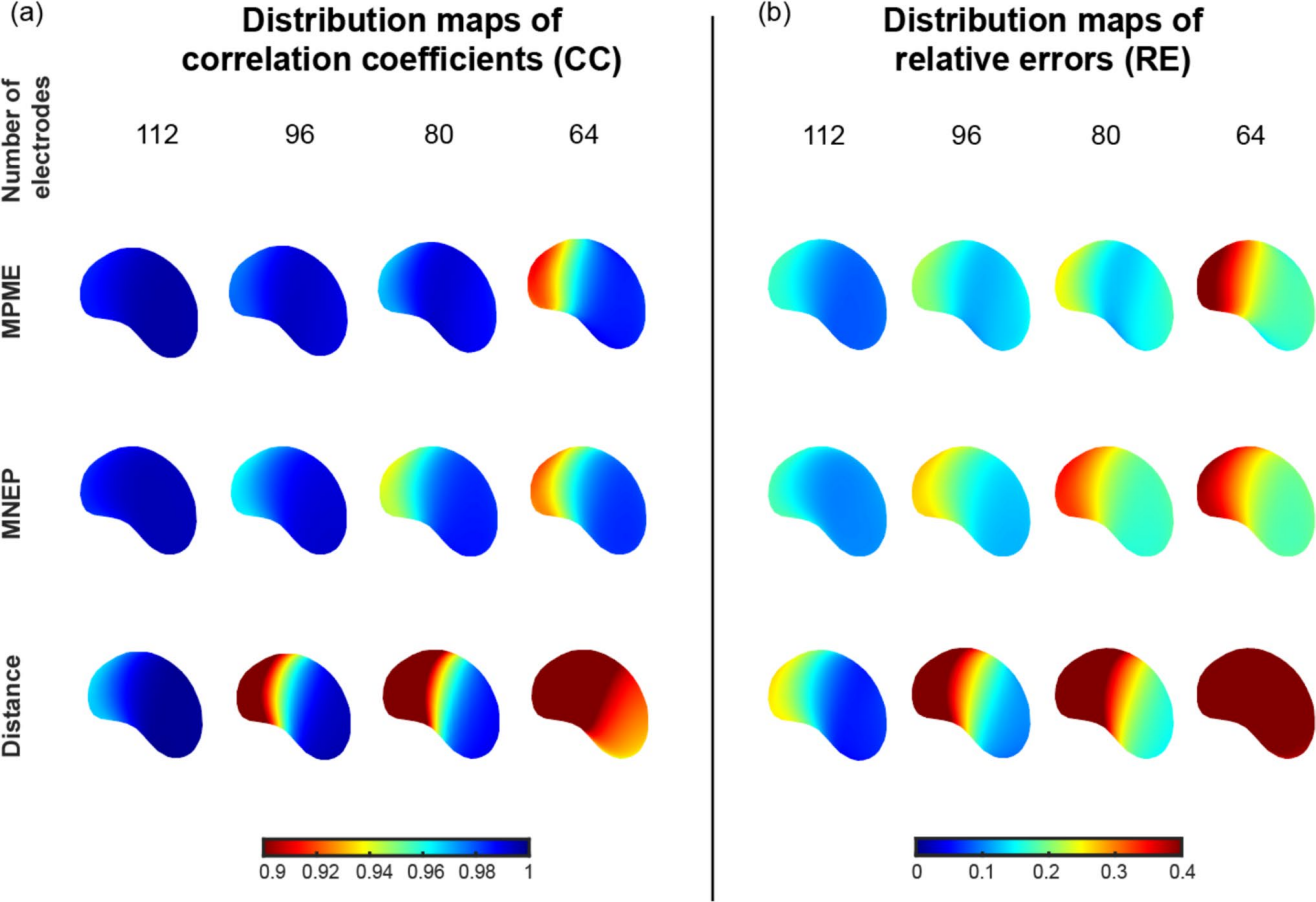
Distribution maps of CC (**a**) and RE (**b**) were generated across the entire uterine surface in subject #1. These maps were created using varying numbers of electrodes (112, 96, 80, and 64) with three electrode optimization methods. CC, correlation coefficient; RE, relative error; MPME, maximal projection on minimum eigenspace; MNEP, minimum nonzero eigenvalue pursuit

For the subject level comparison, Fig. 3 presents the CC and RE distribution maps across the entire uterine surface. In these spatial distribution maps, cool colors indicate regions with high CC and low RE, signifying good accuracy, while warm colors indicate regions with low CC and high RE, signifying poor accuracy. The poor accuracy regions expand as the number of electrodes decreases across all three methods.

**Fig. 4:**
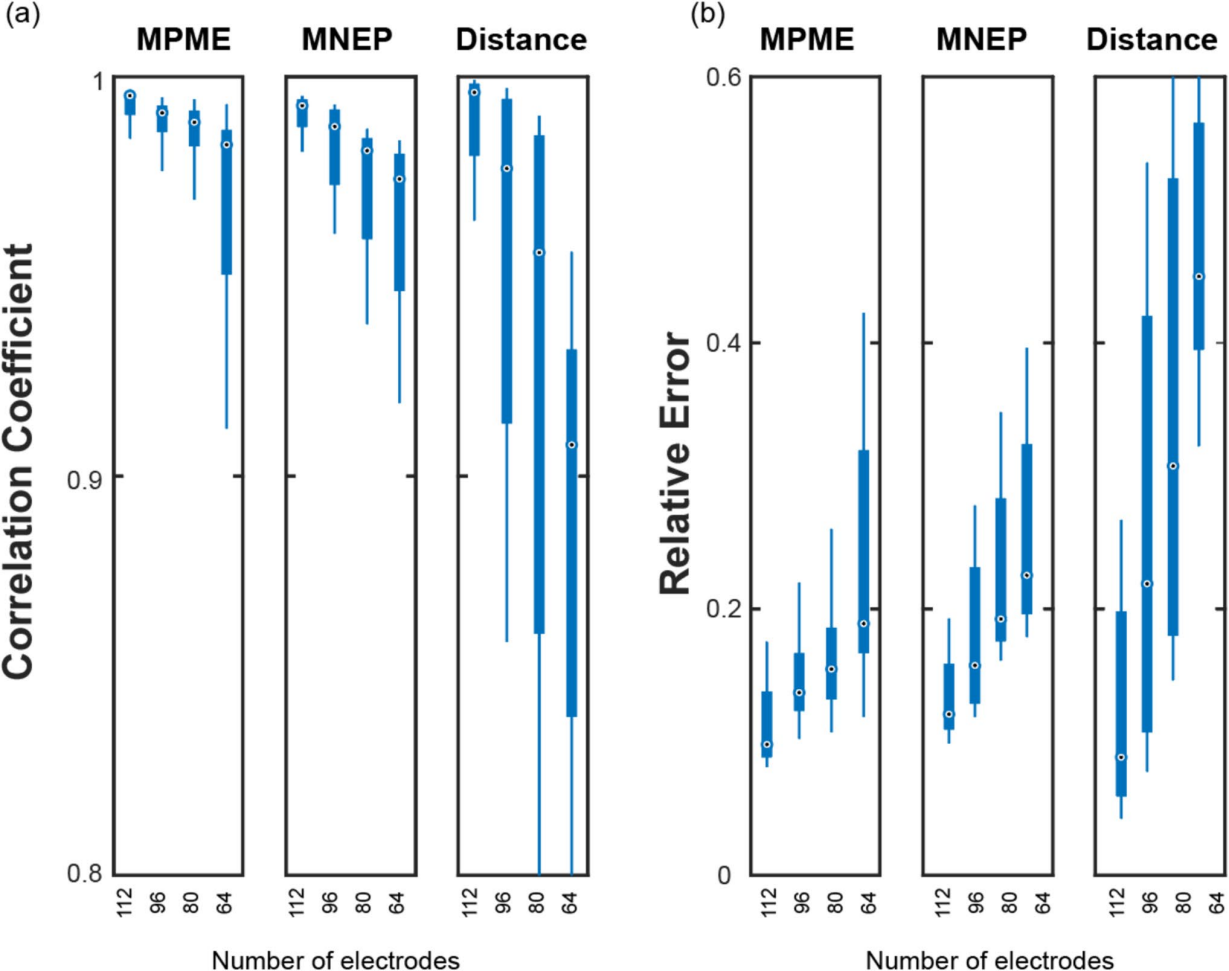
Boxplots of CC (**a**) and RE (**b**) with varying numbers of electrodes in subject #1, comparing the MPME, MNEP, and distance-based method. The entire uterine surface was discretized into 320 uterine sites, and each boxplot summarizes the CC or RE of all 320 uterine sites. The numbers of electrodes were reduced from 112 to 64 in steps of 16. CC, correlation coefficient; RE, relative error; MPME, maximal projection on minimum eigenspace; MNEP, minimum nonzero eigenvalue pursuit

The quantitative results of the subject level are shown in Fig. 4 and Table 2 in the Supplementary section. For all three methods, the mean values of the CC decreased, and the mean values of RE increased as the number of electrodes decreased. The mean values of CC of the MPME method ranged from 0.969 to 0.993 for electrode numbers ranging from 64 to 112. The mean values of the RE of the MPME method ranged from 0.113 to 0.236 for electrode numbers from 112 to 64. The mean values of CC of the MNEP method ranged from 0.963 to 0.991 for electrode numbers from 64 to 112. The mean values of RE of MNEP ranged from 0.134 to 0.259 for electrode numbers from 112 to 64. The mean values of CC of the distance-based method ranged from 0.888 to 0.989 for electrode numbers from 64 to 112. The mean values of RE of the distance-based method ranged from 0.125 to 0.472 for electrode numbers from 112 to 64. The MPME method outperformed the other two methods in all cases in terms of mean CC and mean RE. The LM for the MPME method was consistently the lowest, with values of 4.160 × 10^3^ for 112 electrodes, 4.172 × 10^3^ for 96 electrodes, 4.187 × 10^3^ for 80 electrodes, and 4.203 × 10^3^ for 64 electrodes, compared to the other two methods.

**Fig. 5:**
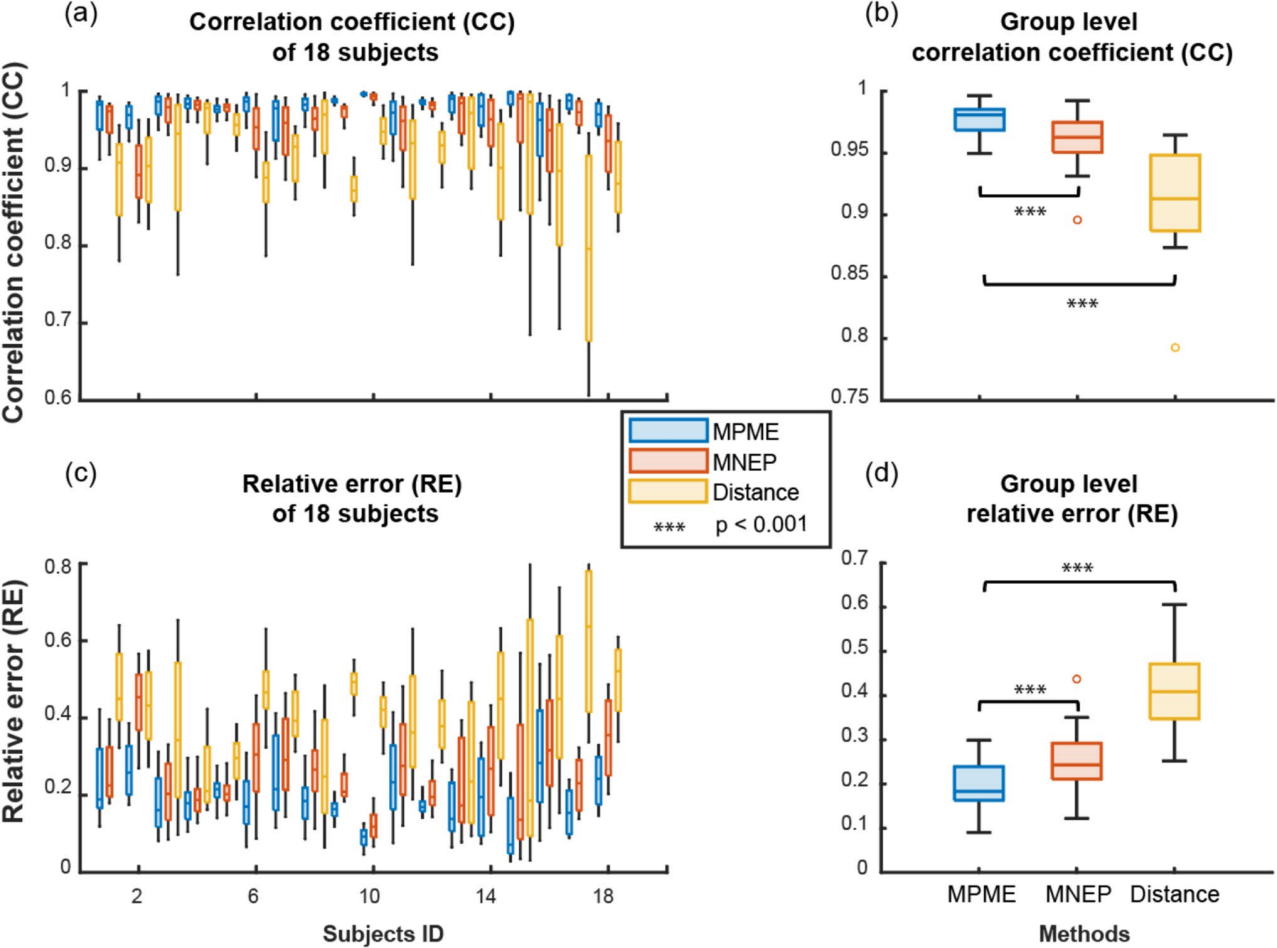
Boxplots of CC and RE between 64-electrode and 128-electrode UPI in 18 subjects at the subject and group level, comparing the MPME, MNEP, and distance-based method. **a** Subject level comparison of CC. **b** Group level comparison of CC. **c** Subject level comparison of RE. **d** Group level comparison of RE. Each dot in the boxplots a and c represents a CC and RE comparison between 64-electrode UPI and 128-electrode UPI on a uterine site. Each dot in the boxplots b and d represents the mean value of CC and RE of each subject. CC, correlation coefficient; RE, relative error; MPME, maximal projection on minimum eigenspace; MNEP, minimum nonzero eigenvalue pursuit; UPI, uterine peristalsis imaging

For the subject level comparison in 18 subjects with 64-electrode UPI compared to 128-electrode UPI, the CC and RE analysis results are shown in boxplots in Fig. 5a, c, as well as Tables 3 and 4 in the Supplementary section. The LM for each subject is shown in Table 5 in the Supplementary section. The mean values of CC of the MPME method ranged from 0.950 to 0.996. The mean values of the RE of the MPME method ranged from 0.090 to 0.299. The LM for the MPME method ranged from 4.040 × 10^3^ to 4.262 × 10^3^. The mean values of the CC of the MNEP method ranged from 0.896 to 0.993. The mean values of the RE of the MNEP method ranged from 0.122 to 0.437. The LM for the MNEP method ranged from 4.054 × 10^3^ to 4.272 × 10^3^. The mean values of the CC of the distance-based method ranged from 0.793 to 0.965. The mean values of the RE of the distance-based method ranged from 0.252 to 0.606. The LM of the distance-based method ranged from 4.079 × 10^3^ to 4.302 × 10^3^. The MPME method achieved the highest mean CC, the lowest LM in 18 subjects, and the lowest mean RE in 17 subjects. The MNEP method achieved the highest mean CC and the lowest mean RE in 1 subject.

For the group-level comparison (Fig. 5b, d), statistical analyses were performed using a Wilcoxon signed-rank test to account for the paired (within-subject) design. The mean values of CC of the MPME method were significantly higher than those of the MNEP and distance-based method with a *p* value = 2.33 × 10^−4^ and *p* value = 1.96 × 10^−4^, respectively. The HL estimate of the median difference was 0.0146 (95% CI [0.0061, 0.0179]) and 0.0670 (95% CI [0.0482, 0.0804]). The corresponding effect sizes were *r* = 0.867 and *r* = 0.878, indicating large effect sizes. The mean values of RE of the MPME method were significantly lower than those of the MNEP and distance-based method, with a *p* value = 2.33 × 10^−4^ and *p* value = 1.96 × 10^−4^, respectively. The HL estimate of the median difference was − 0.0588 (95% CI [− 0.0754, − 0.0318]) and − 0.1975 (95% CI [− 0.2486, − 0.1479]. The corresponding effect sizes were *r* = − 0.867 and *r* = − 0.878, indicating large effect sizes.

### Evaluation of the Prioritized Electrode Locations under the Constraint of the Original UPI Placement

**Fig. 6:**
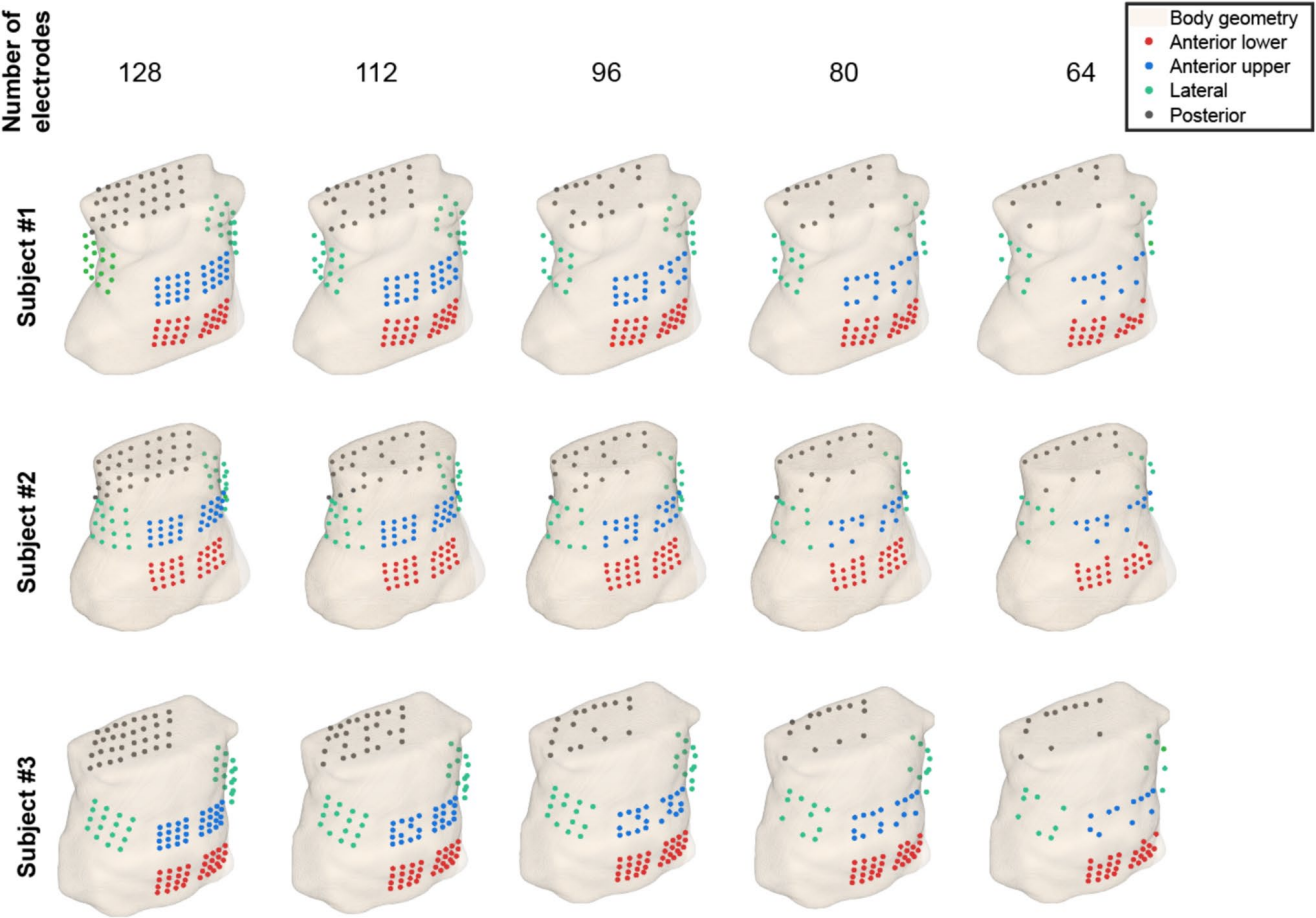
Original 128-electrode placement and electrode placement with varying numbers of electrodes (112, 96, 80, and 64) for three representative subjects using the MPME method. MPME, maximal projection on minimum eigenspace

Spatial configurations of electrode placements for 112, 96, 80, and 64 electrodes were generated using the MPME method for three representative subjects, as shown in Fig. 6. The electrodes were grouped into four regions: anterior upper, anterior lower, lateral, and posterior regions. As the number of electrodes decreased, the MPME method prioritized the anterior lower region over other regions. Within the upper, lateral, and posterior regions, electrodes positioned along the edges, which better preserved the geometric contours, were prioritized over those in central areas. The corresponding subject-specific uterine shapes for these representative cases are shown in the Supplementary Fig. S1 to illustrate the anatomical context.

### Evaluation of the Prioritized Electrode Locations without the Constraint of the Original UPI Placement

**Fig. 7:**
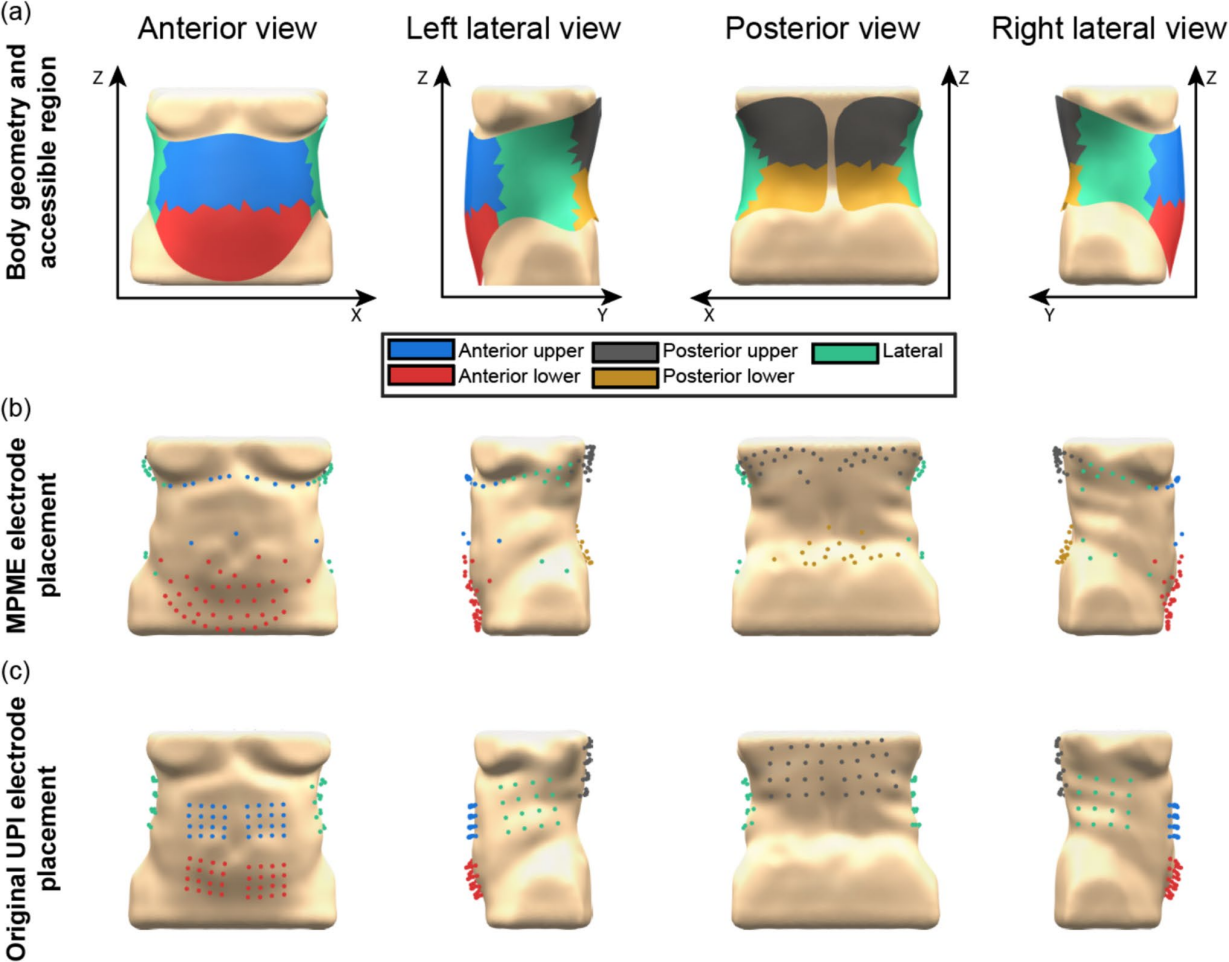
Comparison of the original UPI and MPME-designed electrode placement. **a** Body geometry and accessible regions viewed from the anterior, left lateral, posterior, and right lateral perspectives. The torso surface was divided into five regions: anterior upper (blue), anterior lower (red), posterior upper (dark gray), posterior lower (brown), and lateral (green). The stepped appearance of the regional boundaries reflects the discretization of the surface mesh rather than anatomical irregularity.** b** Electrode configuration optimized using the MPME method. **c** Original UPI electrode placement. MPME, maximal projection on minimum eigenspace; UPI, uterine peristalsis imaging

Compared to the original 128-electrode UPI configuration (Fig. 7c), the MPME-designed layout (Fig. 7b) produced a distinct redistribution of electrodes across the accessible torso surface. In the original layout, electrodes were uniformly distributed across four regions, with 32 electrodes (25.0%) placed in each of the anterior upper, anterior lower, lateral, and posterior upper regions, and none in the posterior lower region.

In contrast, the MPME optimization yielded a non-uniform distribution. The largest proportion of electrodes was allocated to the anterior lower region (41/128, 32.0%), followed by posterior upper (31/128, 24.2%), lateral (25/128, 19.5%), posterior lower (17/128, 13.3%), and anterior upper (14/128, 10.9%). Relative to the original layout, the optimized configuration increased electrode density over the anterior lower region and introduced substantial coverage in the posterior lower region, where no electrodes were previously placed. Additional redistribution occurred along the lower boundaries of the lateral regions while maintaining moderate coverage in the upper posterior region and upper abdomen.

This spatial reallocation was associated with improved conditioning of the system matrix. The LM for the original 128-electrode placement was 4.146 × 10^3^, while the LM for the designed electrode layout was 3.926 × 10^3^. The MPME-designed electrode placement substantially reduced the LM compared to that of the original 128-electrode placement.

## Discussion

This study generalized and evaluated the MPME method in the context of UPI for electrode optimization, demonstrating superior performance in both accuracy and robustness. At both the uterine site and subject levels, the MPME method consistently outperformed the other two methods in terms of CC and RE, effectively preserving the magnitude and morphology of uterine surface electrograms. The MPME method also achieved the lowest LM, indicating that optimizing the LM results in higher CC and lower RE.

For the group level comparison, we employed MRI-derived, subject-specific geometry to specify electrode locations and uterine surface geometry. This approach accounted for the variations in the electrode configurations, as well as differences in uterine shape, location, and orientation. The Wilcoxon signed-rank test indicated that the MPME method achieved significantly higher reconstructed imaging accuracy than the other two methods, confirming its robustness and generalizability. The *p* values for RE and CC are equivalent due to the two sets of paired data having similar ranks of difference. This finding indicates a strong correlation between the quality of reconstruction in terms of magnitude and morphology. Beyond the statistical significance, the effect sizes were large for the MPME method compared to the other two methods, indicating that the improvement in reconstruction accuracy using the MPME method was substantial. Such a strong effect suggests that the optimized electrode placement plays a crucial role in reconstructing the uterine electrophysiological activity and is likely to have a meaningful impact on clinical utility, rather than being a minor technical adjustment.

The rationale behind this improvement lies in the principle of different optimization algorithms. Compared with the distance-based method, which is not a systematic optimization approach, both MNEP and MPME consistently outperform it. The distance-based method tends to preserve only the electrodes on the lower abdomen while neglecting other regions, whereas the optimization-based methods provide broader and more balanced electrode selection.

Compared with the MNEP method, which attempts to maximize the minimum nonzero eigenvalue at each step, the MPME method provides a more straightforward geometric strategy. By maximizing the projection onto the current minimum eigenspace, MPME directly targets the most poorly observed subspace. This bottom-up strengthening systematically repairs the weakest directions, leading to steady improvement of the eigenvalue spectrum and ultimately producing a better-conditioned system. This geometric focus explains why MPME achieves better performance than MNEP.

Importantly, although our cohort included both healthy subjects and subjects with underlying conditions, we did not observe systematic differences in the optimized electrode configurations between these groups. This is expected, because the electrode optimization process is driven by the conditioning of the system matrix, which depends primarily on uterine and torso geometries rather than subject-specific uterine electrical patterns. While electrophysiological activity may vary across clinical groups, the spatial electrode selection itself is determined by forward model characteristics. This finding further supports the robustness and generalizability of the MPME method across diverse physiological conditions.

Importantly, these numerical comparisons provide practical guidance for electrode array design. Under the constraint of the original 128 electrode UPI configuration, the MPME method redistributed electrodes toward the lower abdomen while reducing emphasis on the upper abdomen. At the same time, it preserved a meaningful number of electrodes across the lateral and posterior regions and introduced coverage in the posterior lower area that was not included in the original layout. This redistribution indicates that regions closest to the uterus, particularly the lower abdominal surface, are prioritized, while broader torso coverage remains necessary.

When the optimization was performed without restricting placement to the original UPI configuration, the MPME-designed layout achieved a lower LM compared to the original electrode placement, indicating improved conditioning of the system matrix. Although CC and RE could not be computed directly in this case due to the lack of signal data, our prior analysis demonstrated that reducing LM is associated with improved reconstruction performance. The reduction in LM, therefore, supports the effectiveness of the optimized placement in enhancing system stability.

Together, the constrained and unconstrained analyses consistently suggest that an optimal electrode configuration should increase sensor density over the lower abdomen while maintaining adequate coverage across the upper abdomen, lateral flanks, and posterior surface. Concentrated placement near the uterus improves sensitivity to uterine electrical activity, whereas distributed coverage across other regions preserves geometric balance and contributes to stable inverse reconstruction. This balance between localized sensitivity and global coverage underlies the improved performance observed with the MPME-designed layouts.

Our study’s findings corroborate those of the previous research, which demonstrated that a high-density electrode configuration concentrated near the heart yields the highest correlation with the 252-reference electrode configuration [20]. In contrast to the prior study, which only compared three fixed electrode configurations (62 electrodes surrounding the thorax, 62 concentrated electrodes directly overlaying the heart, and 62 super-concentrated electrodes directly overlaying the heart) without employing a systematic optimization approach, our research addresses the limitations comprehensively. Furthermore, the previous study indicated that utilizing an anterior super-concentrated electrode configuration could not reconstruct the information on the posterior area. To address this issue, our method involved strategically placing electrodes near the organ of interest from different angles, ensuring comprehensive data acquisition and enabling accurate reconstruction of the posterior area.

Our systematic approach to electrode placement aims to enhance the accuracy of the reconstruction. Previous studies have shown that reducing the number of electrodes can still maintain a high level of agreement between the ECGI and Carto in determining the site of origin of the arrhythmia [19]. Specifically, the agreement rate was 85% with 23 electrode bands (143 electrodes), and the agreement rate was 80% when the number of electrodes was reduced by half (73 electrodes). However, this study reduced the electrode number by evenly removing the electrode bands, which is not a systematic approach. Our method, on the other hand, employs a more systematic approach, which is expected to further enhance the accuracy of the reconstruction.

It is important to acknowledge the limitations of this study. First, this study was conducted with a relatively small sample size of 18 subjects, which limits the statistical power and generalizability of the findings. Future investigations should include larger and more diverse cohorts to enhance the robustness of the findings. Second, the MPME is a greedy method that lacks a theoretical guarantee of reaching the global optimum. In addition, the greedy approach involves sequentially removing electrodes, which may not be suitable for all situations, particularly when dynamic adjustment or specific constraints on electrode placement are required [28, 29]. Future research could explore alternative optimization techniques to address the limitations of the greedy approach, developing more flexible strategies for electrode optimization. With the rapid advances in machine learning and deep learning, integrating learning-based electrode optimization and GPU-accelerated inverse solvers could further enhance performance [30, 31]. Third, a practical challenge remains in translating optimized selections into deployable electrode patches. The MPME method often yields irregular spatial configurations, which are difficult to implement in clinical settings, where rapid and consistent deployment is critical. An open question is how to systematically group these irregular selections into a small number of patches. For example, abdominal electrodes could be divided into two semicircular patches, while those along the upper boundary might be assembled into a belt-like array. Prospective clinical translation will require prototype electrode patches and quantitative inter-session reproducibility metrics before patient use. Addressing this issue will be essential for bridging the gap between computational optimization and real-world applications. Last, this study also assumed a homogeneous medium between the uterine and body surfaces. Incorporating layered tissue modeling in the future would likely improve the accuracy of UPI reconstructions [32]. Continued efforts along these directions will improve the accuracy, usability, and clinical applicability of electrophysiological imaging across diverse contexts.

In summary, this study has advanced electrode optimization in UPI applications by identifying critical locations and proposing an optimized electrode placement. Furthermore, the findings suggest a natural extension of applying MPME to other imaging techniques with similar methodologies, such as ECGI and EMMI. These contributions lay a foundation for improving the accuracy of electrophysiology imaging, reducing hardware costs, and benefiting research in the design and fabrication of portable imaging systems, as well as clinical diagnostics.

## Supplementary Information

Below is the link to the electronic supplementary material.

Supplementary file1 (DOCX 439 kb)
