# Supplementary material for: Optimization of the Multi-Channel Surface Electrogram Acquisition for Noninvasive Uterine Electrophysiology Imaging
Source: Ann Biomed Eng. 2026 Apr 10;54(8):2748–62. doi: 10.1007/s10439-026-04072-2 (PMC13391704; doi:10.1007/s10439-026-04072-2)
Supplement: Supplementary file 1 — Supplementary file1 (DOCX 439 kb) [file 10439_2026_4072_MOESM1_ESM.docx]

# Supplementary

**Electrode Placement**

Electrodes were placed with the subject in a standing position.

For posterior placement, the end of the tailbone was first identified, and the base of a 30-cm vertical ruler was aligned with this landmark along the spine. Electrode patches were then positioned bilaterally at the lower end of the ruler.

For anterior and lateral placement, the vertical ruler was placed at the start of the pubic region. The lower set of electrode patches was placed bilaterally at the base of the ruler. A horizontal ruler was then aligned with the upper edge of these patches, and the next set of patches was positioned directly above the ruler. Finally, the lateral patches were placed above the horizontal ruler, ensuring symmetric positioning on both sides.

**Forward Computation**

For the forward problem of the UPI system, it is assumed that there is a homogeneous medium between the uterine and body surfaces without any primary electrical source [33, 34]. The bioelectric field reconstruction can be mathematically described by solving the Cauchy problem for Laplace’s equation (1) with boundary conditions (2,3) on the body surface:

$$\begin{aligned} \nabla^{2}\phi\left( x \right)=0\#\left( 9 \right) \end{aligned}$$

Dirichlet (2) and Neumann (3) conditions for the body surface potentials are

$\begin{aligned} \phi\left( x \right)=\phi_{B\left( x \right)}, x\in\Gamma_{B}\#\left( 10 \right) \end{aligned}$

$\begin{aligned} \frac{\partial\phi\left( x \right)}{\partial n}=0, x\in\Gamma_{B}\#\left( 11 \right) \end{aligned}$

Here, $\phi\left( x \right)$ represents the bioelectric field, $\Gamma_{B}$ is the body surface, $n$ is the normal vector on the body surface at $x$, and $\phi_{B\left( x \right)}$ is the potential measured on the body surface.

The method of fundamental solutions (MFS) is a mesh-free method known for its robustness to noise. It is used to discretize Laplace’s equation and boundary conditions that have been proven highly accurate in solving the inverse problem related to the bioelectric field:

$$\begin{aligned} \phi_{B}={A \phi}_{U}\#\left( 12 \right) \end{aligned}$$

where $= \left[ \begin{matrix} \begin{matrix} f(\|x_{1}-y_{1}\|) \\ \cdots\\ f(\|x_{N}-y_{1}\|) \end{matrix} & \begin{matrix} \cdots& f(\|x_{1}-y_{M}\|) \\ \cdots& \cdots\\ \cdots& f(\|x_{N}-y_{M}\|) \end{matrix} \\ \begin{matrix} \frac{\partial f(\|x_{1}-y_{1}\|)}{\partial n} \\ \cdots\\ \frac{\partial f(\|x_{N}-y_{1}\|)}{\partial n} \end{matrix} & \begin{matrix} \cdots& \frac{\partial f(\|x_{1}-y_{M}\|)}{\partial n} \\ \cdots& \cdots\\ \cdots& \frac{\partial f(\|x_{N}-y_{M}\|)}{\partial n} \end{matrix} \end{matrix} \right]$ , $\phi_{B}=\left[ \begin{matrix} \begin{matrix} \phi_{11} & \ldots& \phi_{1T} \\ \vdots& \ldots& \vdots\\ \phi_{N1} & \ldots& \phi_{NT} \end{matrix} \\ \begin{matrix} 0 & \ldots& 0 \\ \vdots& \ldots& \vdots\\ 0 & \ldots& 0 \end{matrix} \end{matrix} \right]$ ,

$\phi_{U}=\left[ \begin{matrix} \phi_{11} & \cdots& \phi_{1T} \\ \vdots& \cdots& \vdots\\ \phi_{M1} & \cdots& \phi_{MT} \end{matrix} \right]$

Matrix $A$ is of dimension $2N\times M$; Matrix $\phi_{B}$ is of dimension $2N\times T$, Matrix $\phi_{U}$ is of dimension $M\times T$, $f\left( r \right)= \frac{1}{4\pi r}$is the fundamental solution for Laplace’s equation, and $r=|\left| x-y \right||$ is the Euclidean distance between point $x$ and $y$.

**Underdetermined Linear Inverse Problem**

We consider that a physical field at a single timepoint on the subject's body surface can be observed from electrode networks, represented as

$\begin{aligned} \phi_{Bt}=A\phi_{Ut}+v\#\left( 13 \right) \end{aligned}$

Here, $\phi_{Bt}\in\mathbb{R}^{2N\times1}$ is the electrical measurement at a single timepoint on the subject's body surface (a column of $\phi_{B}$), $\phi_{Ut}\in\mathbb{R}^{M\times1}$ is the uterine surface potential (a column of $\phi_{U}$), $A\in\mathbb{R}^{2N\times M}$ is the system matrix$,$ and $v\in\mathbb{R}^{2N\times1}$ is the measurement noise on the body surface, assumed to be zero-mean i.i.d Gaussian noise with variance $\sigma^{2}I${Citation}.

If matrix A has more rows than columns, the minimum variance unbiased estimate (MVUE) is given by [23]

$\begin{aligned} \hat{\phi_{Ut}}=A^{+}\phi_{Bt}\#\left( 14 \right) \end{aligned}$

where $A^{+}=\left( A^{T}A \right)^{-1}A^{T}$ is the pseudo-inverse of $A$. Therefore, the mean square error (MSE) of this MVUE is

$\begin{aligned} MSE\left( \hat{\phi_{Ut}} \right)=E\left( \left\| \hat{\phi_{Ut}}-\phi_{Ut} \right\|_{2}^{2} \right)=\sigma^{2}tr \left( \Psi^{-1} \right)=\sigma^{2}\sum_{k=1}^{n} \frac{1}{\lambda_{k}}\#\left( 15 \right) \end{aligned}$

where $\lambda_{1}\geq\lambda_{2}\geq\ldots\lambda_{n}$ are the eigenvalues of $\Psi=A^{T}A$

However, for the typical UPI setting, the imaging parameters $M$ are larger than the discretized boundary conditions $2N$. The problem is underdetermined and cannot be solved directly by inverting matrix *A*. We used a well-established technique, Tikhonov regularization, to solve the underdetermined linear inverse problem. Instead of just minimizing $\left\| A\phi_{Ut}-\phi_{Bt} \right\|$, we minimize the cost function:

$\begin{aligned} \left\| A\phi_{Ut}-\phi_{Bt} \right\|^{2}+\alpha^{2}\left\| \phi_{Ut} \right\|^{2}\#\left( 16 \right) \end{aligned}$

We denote

$\begin{aligned} L\left( s \right)=tr \left( \left( \Psi+\alpha I \right)^{-1} \right)=\sum_{k=1}^{n} \frac{1}{\lambda_{rk}}\#\left( 17 \right) \end{aligned}$

as the loss metric (LM), where $s$ represents the selected set of electrodes and $\alpha$ is a small constant ($\alpha$ = 0.1 in this study), $\lambda_{r1}\geq\lambda_{r2}\geq\ldots\lambda_{rn}$ are the eigenvalues of $\Psi+\alpha I$. Adding this term can solve the problem of rank deficiency. After adding the regularization term, the eigenvalues of $\Psi=A^{T}A$ are added by $\alpha^{2}$ to get the eigenvalues of $\Psi+\alpha I$. To minimize the LM, we need to optimize both the quantity and magnitude of the nonzero eigenvalues of $\Psi$, which is determined by the electrode sensing locations.

**Subject-Specific Uterine Geometry and Its Influence on Electrode Layout**

Fig. S1 shows the subject-specific uterine geometries for the three representative subjects presented in Fig. 6. For each subject, the uterus is displayed within the body geometry (left) and in isolated coronal, sagittal, and axial views (right). Although moderate anatomical variability in uterine shape is observed across subjects, the optimized electrode layouts in Fig. 6 remain spatially consistent. This demonstrates that small differences in uterine geometry do not materially affect the overall electrode placement pattern.

**
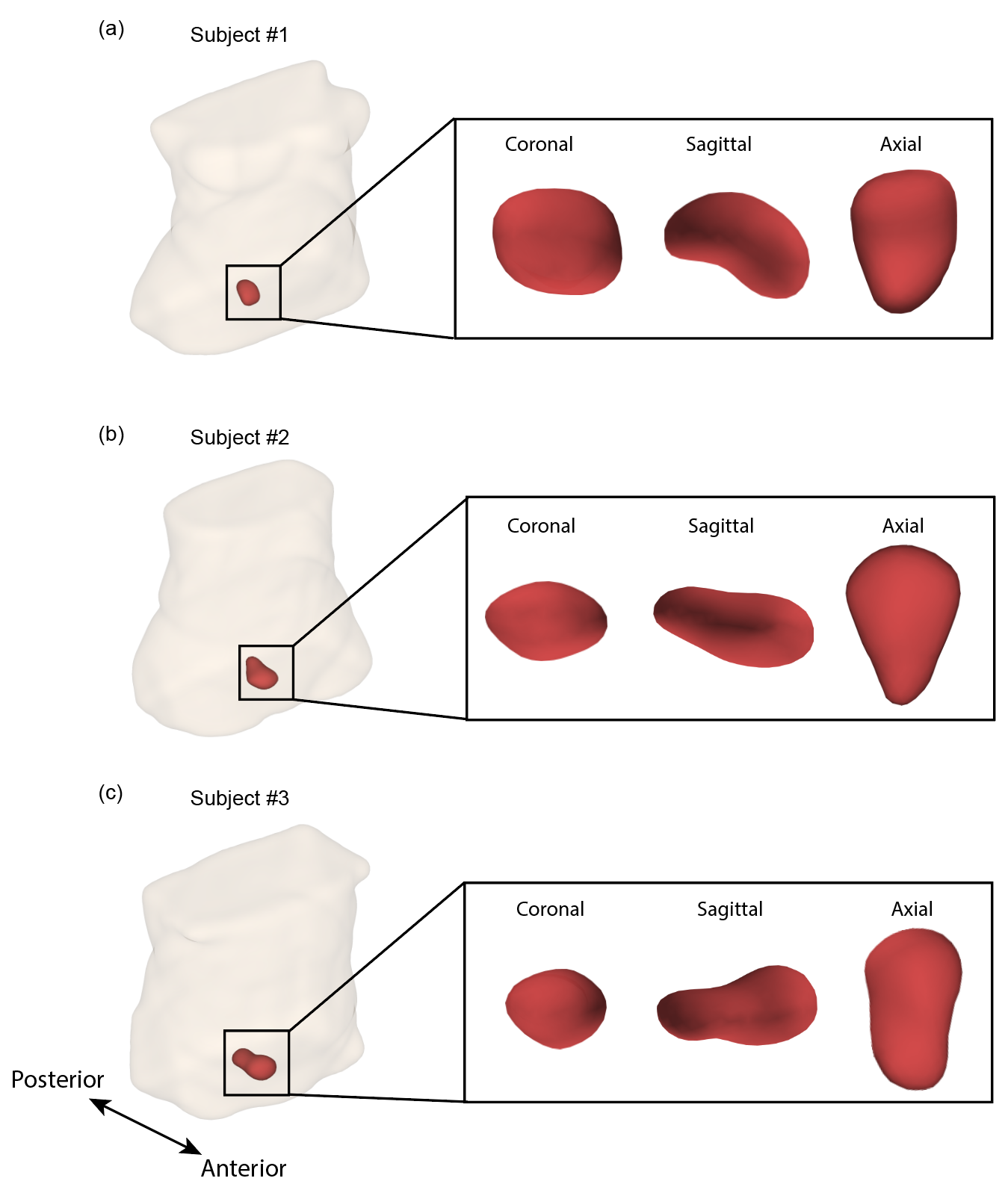
Fig S1** Subject-specific uterine geometries for three representative subjects used in Fig. 6. For each subject, the uterus is shown within the body geometry (left) and in isolated views (right), including coronal, sagittal, and axial projections

| Methods | 112 electrodes | 96 electrodes | 80 electrodes | 64 electrodes |
| --- | --- | --- | --- | --- |
| MPME CC | **0.993 ± 0.004** | **0.989 ± 0.005** | **0.986 ± 0.008** | **0.969 ± 0.025** |
| MNEP CC | 0.991 ± 0.004 | 0.983 ± 0.011 | 0.972 ± 0.016 | 0.963 ± 0.022 |
| Distance CC | 0.989 ± 0.012 | 0.954 ± 0.047 | 0.923 ± 0.071 | 0.888 ± 0.054 |
| MPME RE | **0.113 ± 0.031** | **0.148 ± 0.032** | **0.166 ± 0.042** | **0.236 ± 0.095** |
| MNEP RE | 0.134 ± 0.029 | 0.178 ± 0.055 | 0.226 ± 0.062 | 0.259 ± 0.073 |
| Distance RE | 0.125 ± 0.077 | 0.263 ± 0.160 | 0.350 ± 0.176 | 0.472 ± 0.096 |
| MPME LM | **4.160****×****10^3^** | **4.172×10^3^** | **4.187×10^3^** | **4.203×10^3^** |
| MNEP LM | 4.162×10^3^ | 4.179×10^3^ | 4.197×10^3^ | 4.214×10^3^ |
| Distance LM | 4.179×10^3^ | 4.207×10^3^ | 4.229×10^3^ | 4.253×10^3^ |

**Table 2** Mean value and standard deviation of CC and RE, and the LM with varying numbers of electrodes in subject #1. The bold fonts indicate the highest CC, lowest RE, and lowest LM. CC, correlation coefficient; RE, relative error; LM, loss metric; MPME, maximal projection on minimum eigenspace; MNEP, minimum nonzero eigenvalue pursuit

| **Subjects** | **MPME CC** | **MNEP CC** | **Distance CC** |
| --- | --- | --- | --- |
| #1 | **0.969 ± 0.025** | 0.963 ± 0.022 | 0.888 ± 0.054 |
| #2 | **0.966 ± 0.016** | 0.896 ± 0.039 | 0.898 ± 0.045 |
| #3 | **0.981 ± 0.015** | 0.975 ± 0.017 | 0.915 ± 0.075 |
| #4 | **0.983 ± 0.009** | 0.981 ± 0.009 | 0.965 ± 0.025 |
| #5 | **0.977 ± 0.006** | **0.977 ± 0.009** | 0.956 ± 0.016 |
| #6 | **0.983 ± 0.012** | 0.951 ± 0.032 | 0.881 ± 0.038 |
| #7 | **0.965 ± 0.026** | 0.950 ± 0.034 | 0.917 ± 0.030 |
| #8 | **0.981 ± 0.012** | 0.962 ± 0.020 | 0.953 ± 0.041 |
| #9 | **0.988 ± 0.003** | 0.973 ± 0.009 | 0.874 ± 0.019 |
| #10 | **0.996 ± 0.001** | 0.993 ± 0.004 | 0.948 ± 0.020 |
| #11 | **0.964 ± 0.026** | 0.951 ± 0.034 | 0.911 ± 0.060 |
| #12 | **0.985 ± 0.004** | 0.981 ± 0.006 | 0.926 ± 0.024 |
| #13 | **0.985 ± 0.011** | 0.971 ± 0.023 | 0.950 ± 0.045 |
| #14 | **0.976 ± 0.020** | 0.958 ± 0.031 | 0.894 ± 0.063 |
| #15 | **0.991 ± 0.011** | 0.961 ± 0.052 | 0.917 ± 0.109 |
| #16 | **0.950 ± 0.041** | 0.934 ± 0.049 | 0.876 ± 0.088 |
| #17 | **0.986 ± 0.009** | 0.971 ± 0.015 | 0.793 ± 0.118 |
| #18 | **0.969 ± 0.015** | 0.931 ± 0.037 | 0.887 ± 0.046 |

**Table** **3** Mean values and standard deviations of CC between 64-electrode UPI and 128-electrode UPI for MPME, MNEP, and the distance-based method in 18 subjects. The bold fonts indicate the highest mean correlation coefficients. CC, correlation coefficient; MPME, maximal projection on minimum eigenspace; MNEP, minimum nonzero eigenvalue pursuit

| **Subjects** | **MPME RE** | **MNEP RE** | **Distance RE** |
| --- | --- | --- | --- |
| #1 | **0.236 ± 0.095** | 0.259 ± 0.073 | 0.472 ± 0.096 |
| #2 | **0.266 ± 0.067** | 0.437 ± 0.086 | 0.432 ± 0.095 |
| #3 | **0.180 ± 0.071** | 0.210 ± 0.077 | 0.362 ± 0.181 |
| #4 | **0.180 ± 0.048** | 0.192 ± 0.041 | 0.252 ± 0.084 |
| #5 | 0.213 ± 0.029 | **0.211 ± 0.037** | 0.290 ± 0.053 |
| #6 | **0.178 ± 0.067** | 0.292 ± 0.108 | 0.473 ± 0.072 |
| #7 | **0.248 ± 0.095** | 0.302 ± 0.097 | 0.404 ± 0.060 |
| #8 | **0.186 ± 0.057** | 0.266 ± 0.078 | 0.269 ± 0.134 |
| #9 | **0.163 ± 0.023** | 0.227 ± 0.037 | 0.488 ± 0.036 |
| #10 | **0.090 ± 0.023** | 0.122 ± 0.034 | 0.413 ± 0.050 |
| #11 | **0.246 ± 0.096** | 0.292 ± 0.104 | 0.387 ± 0.130 |
| #12 | **0.174 ± 0.021** | 0.206 ± 0.039 | 0.387 ± 0.069 |
| #13 | **0.161 ± 0.062** | 0.224 ± 0.108 | 0.273 ± 0.150 |
| #14 | **0.197 ± 0.095** | 0.265 ± 0.114 | 0.436 ± 0.137 |
| #15 | **0.112 ± 0.080** | 0.227 ± 0.182 | 0.347 ± 0.305 |
| #16 | **0.299 ± 0.134** | 0.332 ± 0.130 | 0.454 ± 0.173 |
| #17 | **0.157 ± 0.055** | 0.228 ± 0.064 | 0.606 ± 0.179 |
| #18 | **0.239 ± 0.062** | 0.351 ± 0.098 | 0.498 ± 0.085 |

**Table 4** Mean values and standard deviations of RE between 64-electrode UPI and 128-electrode UPI for MPME, MNEP, and distance-based method in 18 subjects. The bold fonts indicate the lowest mean relative errors. RE, relative error; MPME, maximal projection on minimum eigenspace; MNEP, minimum nonzero eigenvalue pursuit

| **Subjects** | **MPME LM** | **MNEP LM** | **Distance LM** |
| --- | --- | --- | --- |
| #1 | **4.203×10^3^** | 4.214×10^3^ | 4.253×10^3^ |
| #2 | **4.235×10^3^** | 4.246×10^3^ | 4.283×10^3^ |
| #3 | **4.185×10^3^** | 4.189×10^3^ | 4.239×10^3^ |
| #4 | **4.112×10^3^** | 4.119×10^3^ | 4.151×10^3^ |
| #5 | **4.131×10^3^** | 4.146×10^3^ | 4.177×10^3^ |
| #6 | **4.229×10^3^** | 4.247×10^3^ | 4.279×10^3^ |
| #7 | **4.200×10^3^** | 4.215×10^3^ | 4.238×10^3^ |
| #8 | **4.104×10^3^** | 4.111×10^3^ | 4.147×10^3^ |
| #9 | **4.247×10^3^** | 4.257×10^3^ | 4.289×10^3^ |
| #10 | **4.167×10^3^** | 4.176×10^3^ | 4.210×10^3^ |
| #11 | **4.110×10^3^** | 4.121×10^3^ | 4.148×10^3^ |
| #12 | **4.237×10^3^** | 4.243×10^3^ | 4.283×10^3^ |
| #13 | **4.117×10^3^** | 4.126×10^3^ | 4.154×10^3^ |
| #14 | **4.216×10^3^** | 4.231×10^3^ | 4.268×10^3^ |
| #15 | **4.040×10^3^** | 4.054×10^3^ | 4.079×10^3^ |
| #16 | **4.127×10^3^** | 4.134×10^3^ | 4.158×10^3^ |
| #17 | **4.218×10^3^** | 4.234×10^3^ | 4.263×10^3^ |
| #18 | **4.262×10^3^** | 4.272×10^3^ | 4.302×10^3^ |

**Table 5** Loss metrics for MPME, MNEP, and the distance-based method in 18 subjects. The bold fonts indicate the lowest LM. LM, loss metric; MPME, maximal projection on minimum eigenspace; MNEP, minimum nonzero eigenvalue pursuit
